# Mutual Regulation of Cardiovascular and Hematopoietic Development

**DOI:** 10.1007/s11886-025-02236-5

**Published:** 2025-04-22

**Authors:** Norika Liu, Haruko Nakano, Atsushi Nakano

**Affiliations:** 1 International Research Center for Medical Sciences, Kumamoto University, 2-2-1 Honjyo, Chuou-ku, Kumamoto-Shi, Kumamoto, 860-0811 Japan; 2 Department of Molecular Cell and Developmental Biology, University of California Los Angeles, Los Angeles, USA; 3 Department of Cell Physiology, The Jikei University School of Medicine, Tokyo, Japan; 4 David Geffen Department of Medicine, Division of Cardiology, University of California Los Angeles, Los Angeles, USA; 5 Eli and Edythe Broad Center of Regenerative Medicine and Stem Cell Research, University of California Los Angeles, Los Angeles, USA; 6 Molecular Biology Institute, University of California Los Angeles, Los Angeles, USA

**Keywords:** Endocardium, Hematopoiesis, Endocardial Cushion, Cardiac Development, Cardiac Macrophage

## Abstract

**Purpose of Review:**

The cardiovascular and hematopoietic systems share molecular mechanisms and regulatory interactions across species. Endocardial hematopoiesis, a debated topic in mice, is actually an evolutionarily conserved process from *Drosophila*. This review explores the origins and significance of endocardial hematopoiesis, highlighting its role in cardiac development and macrophage formation.

**Recent Findings:**

Despite extensive lineage-tracing and transcriptome studies, it remained unclear until single-cell RNA sequencing (scRNA-seq) identified that endocardial cells possess an intrinsic hematopoietic program independent of known hematopoietic organs. These endocardial-derived macrophages contribute uniquely to cardiac morphogenesis, supporting valve maturation and tissue remodeling.

**Summary:**

Endocardial hematopoiesis is an evolutionarily conserved phenomenon that is essential for developmental process. The heterogeneity of tissue-resident macrophages and their specialized functions in cardiac development have been further unraveled by single-cell analysis. This review provides an evolutionary perspective on endocardial hematopoiesis and highlights its critical contributions of hematopoietic cells to heart formation and homeostasis.

## Introduction

Hematopoiesis and cardiogenesis are among the earliest developmental processes in vertebrates, forming the foundation of the circulatory system. In mammals, embryonic hematopoiesis occurs in multiple waves across extra- and intra-embryonic sites [1]. The first wave, known as primitive hematopoiesis, arises in the yolk sac blood islands, producing erythromyeloid progenitors (EMPs) that generate primitive erythrocytes and macrophages [2, 3]. A subsequent wave originates from hemogenic endothelial cells in the yolk sac, generating late-EMPs that migrate to the fetal liver, where they expand and differentiate into multiple hematopoietic lineages, including monocytes [4]. Concurrently, hematopoietic progenitors also emerge from intraembryonic hemogenic endothelium in the aorta-gonad-mesonephros (AGM) region, giving rise to definitive hematopoietic stem cells (HSCs) [5].

In addition to these well-characterized hematopoietic sites, recent studies have revealed that endocardial cells in the developing heart possess transient hematopoietic potential, establishing the heart as a previously unrecognized fetal hematopoietic niche. While hematopoiesis within the heart was first described in *Drosophila* larvae [6–8], where the cardiac tube functions as the primary site of blood cell production, its existence in vertebrates has only recently gained attention [9–14]. The evolutionary conservation of this process suggests that endocardial hematopoiesis represents an ancestral mechanism retained across species.

Despite its evolutionary significance, endocardial hematopoiesis has long been overlooked in mammals, likely due to its minor contribution to total blood production. However, recent findings indicate that endocardial-derived hematopoietic progenitors give rise to macrophages that play a unique and indispensable role in cardiac morphogenesis—functions that cannot be compensated for by macrophages originating from other hematopoietic sources [10, 13]. These discoveries have reshaped our understanding of hematopoiesis within the developing heart, highlighting its qualitative rather than quantitative importance.

Nevertheless, the dynamic nature of hematopoietic cells has made it challenging to definitively establish the presence, extent, and physiological relevance of endocardial hematopoiesis. Cre-based lineage tracing studies have struggled to capture this transient and spatially restricted process [15, 16]. However, the use of scRNA-seq has provided unprecedented resolution and revealed that endocardial cells possess an intrinsic hematopoietic program independent of other known hematopoietic organs [9, 13]. Emerging evidence from zebrafish models further supports the concept of endocardial hematopoiesis as a conserved phenomenon, reinforcing its developmental significance across species [17, 18].

Here we summarize the current knowledge on endocardial hematopoiesis, focusing on the conservation across species, the molecular mechanisms involved, its significance in vivo, as well as the challenges and future perspectives. Beyond endocardial hematopoiesis, this review summarizes studies showing the contribution of hematopoietic cells in cardiac development, providing new insights into the complexity of cardiogenesis.

### Cardiogenic mesoderm-derived Hematopoiesis in *Drosophila*

**Fig. 1:**
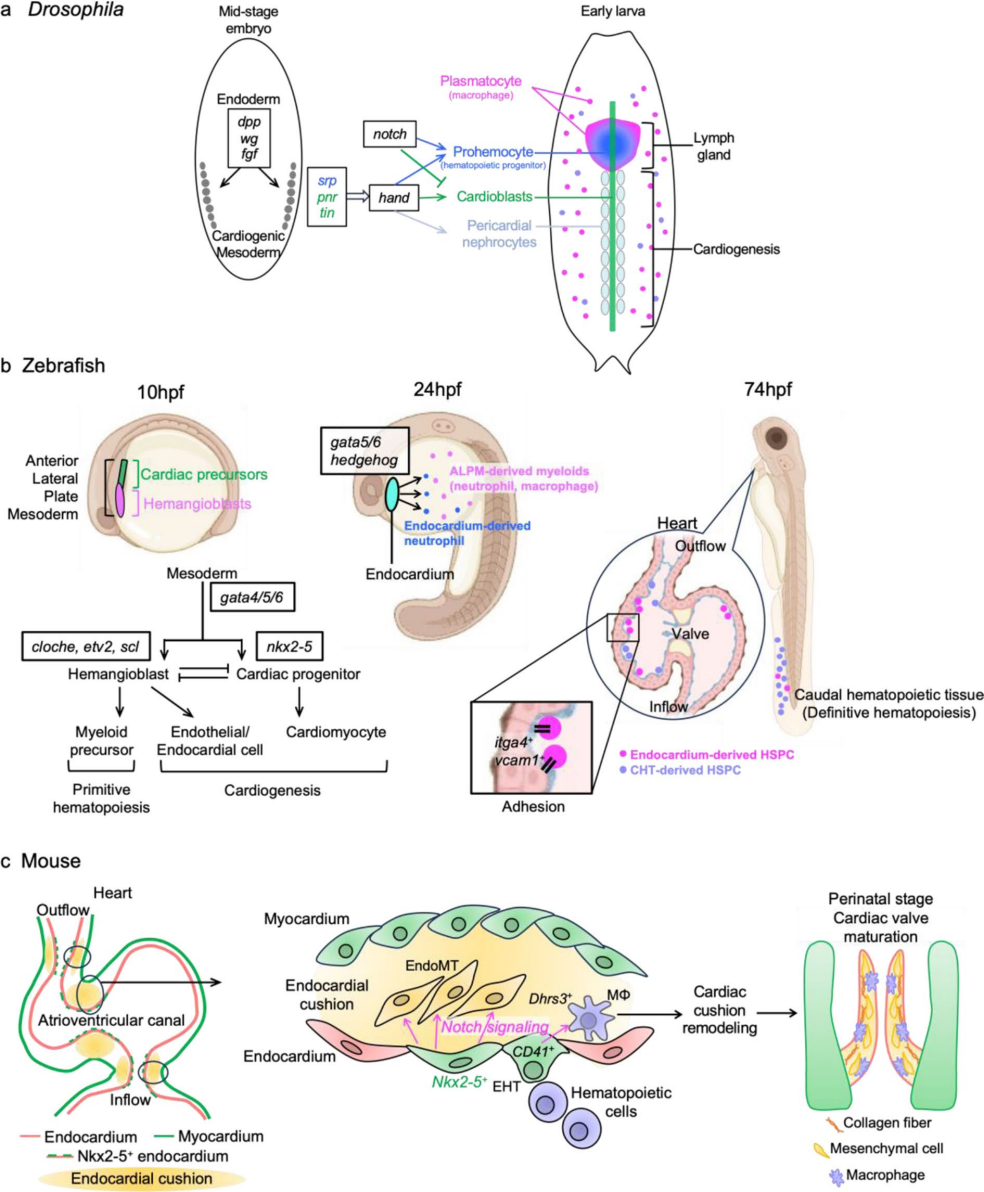
Evolutionarily conserved mechanisms of common cardio-hematopoietic specification. (**a**) *Drosophila* cardiogenic mesoderm is the common origin of lymph gland hematopoietic progenitors, cardioblasts, and pericardial nephrocytes. The dynamic interaction between *notch* signaling and transcription factors such as *srp*,* pnr*,* tin*, and *hand* is essential for guiding the correct lineage commitment. (**b**) Embryonic hematopoiesis in zebrafish occurs in the ALPM, which serves as a common origin for both cardiac precursors and hemangioblasts during early embryonic stages. Recent studies have identified endocardial hematopoiesis around 24 hpf, primarily giving rise to neutrophils [16]. In later stages, endocardial hematopoiesis is also observed concurrently with definitive hematopoiesis, contributing to the formation of HSPCs [17]. (**c**) During mouse cardiogenesis, SHF-derived *Nkx2-5*-lineage endocardial cells undergo EHT through *Notch* signaling activation [6, 7]. Hemogenic endocardial cells (CD41^+^) differentiate into cardiac tissue macrophages (MΦs) by the inhibition of RA signaling through *Dhrs3* expression, contributing to cardiac cushion remodeling and, consequently, valve maturation [7, 10]. Created in BioRender. Liu, N. (2025) https://BioRender.com/f04y383, and are partly adapted from Liu N and Nakano A, Front. Physiol. 2024 [14], under the terms of the Creative Commons Attribution License (CC BY)

In *Drosophila*, signaling pathways involving *bone morphogenetic protein (bmp)*,* decapentaplegic (dpp)*,* wingless (wg*,* Wnt1* homolog), and *fibroblast growth factor (fgf)* are essential for specifying the cardiac mesoderm, the common origin of cardioblasts, pericardial nephrocytes, and lymph gland hematopoietic progenitor cells [19, 20]. This developmental framework suggested a potential link between cardiogenesis and hematopoiesis, laying the groundwork for understanding how these processes are interconnected (Fig. 1a). While such relationships had been postulated, direct in vivo evidence demonstrating how a single progenitor cell divides into both blood and endothelial cells in vertebrates was lacking for a long time [5, 21–23].

Seminal studies in *Drosophila* larvae provided critical insights into the connection of cardiogenesis and hematopoiesis. Using the FLP/FRT system to trace labeled clones, researchers demonstrated that the cardiogenic mesoderm contributes to both lymph gland prohemocytes and cardioblasts [6]. This discovery was pivotal in identifying the dual potential of the cardiogenic mesoderm, a concept that would later resonate in vertebrate systems.

One of the key findings was the role of signaling pathways, particularly *wg* and *notch*, in regulating the differentiation of the cardiogenic mesoderm. *Wg* signaling promotes the differentiation of the mesoderm into both lymph gland and cardioblast lineages, whereas *notch* signaling acts as an inhibitor [6]. Notably, *notch* signaling functions in a time-dependent manner: early in development, it restricts the cardiogenic mesoderm, while later, it facilitates the production of lymph gland prohemocytes and reduces the formation of cardioblasts [6]. This temporal regulation highlights the complexity of mesodermal fate determination and suggests a fine-tuned molecular mechanism that ensures proper differentiation. At the molecular level, *notch* signaling regulates the expression of *serpent* (*srp*), a GATA transcription factor crucial for lymph gland differentiation [6]. In contrast, restricting *notch* signaling maintains the expression of *tinman* (*tin*,* Nkx2-5* homolog) and *pannier* (*pnr*), key factors driving cardioblast formation [6]. This dynamic interaction between signaling pathways and transcription factors is essential for guiding the correct lineage commitment. The link between cardiogenesis and hematopoiesis is further reinforced by the discovery that the basic helix-loop-helix (bHLH) transcription factor *hand* acts as a common downstream target of both *srp* and *tin* [7]. This close lineage relationship between cardiognesis and hematopoiesis and the genetic regulatory mechanisms involved have been validated by more refined and recent lineage tracing experiments and scRNA-seq analyses [24, 25].

The conservation of these processes across species is further supported by the recent discovery of endocardial hematopoiesis in vertebrates. In species ranging from zebrafish to mammals, hematopoietic progenitors are derived from endocardium, a tissue derived from the cardiogenic mesoderm. This finding provides a direct link between cardiac progenitors and hematopoietic cells, mirroring the findings in *Drosophila*. The evolutionary conservation of this relationship suggests that the mechanisms governing the interaction between these two lineages are deeply rooted and likely played a critical role in the evolution of vertebrate circulatory systems.

### Embryonic Hematopoiesis in Zebrafish

Like the cardiogenic mesoderm in *Drosophila*, the ALPM in zebrafish harbors progenitors for both cardiac and hematopoietic lineages [26–29]. Within the ALPM, the rostral region contains hemangioblasts that give rise to hematopoietic, endothelial, and endocardial cells [30–32], while the adjacent posterior region comprises cardiac precursors that differentiate into cardiomyocytes [33, 34]. This spatial organization underscores the distinct gene regulatory programs that govern hemangioblast and cardiac progenitors, while also revealing their interplay (Fig. 1b). For example, mutant embryos of *cloche*, a gene essential for hematopoietic and endothelial specification [35], result in ectopic cardiomyocyte formation in the rostral region of the ALPM [36]. Likewise, knockout mice of hematopoietic transcription factor *Scl/Tal1* activated cariac progenitor transcription in endothelial/endocardial cell lineages [37]. Moreover, transcription factors such as *gata4*, *gata5*, and *gata6* act upstream of shared lineage-specific programs, highlighting the latent cardiogenic potential within the hemangioblast region [28, 30, 36, 38–40]. These findings suggest that the rostral ALMP region represents an evolutionary precursor to the mammalian secondary heart field (SHF), which is known to contribute to the outflow tract and arterial pole of the heart [28].

In zebrafish, SHF progenitors originating from the ALPM require conserved gene expression programs, including *nkx2-5*, which parallels mammalian SHF development [41]. Interestingly, the endocardium derived from the rostral ALPM not only contributes to cardiac morphogenesis but also exhibits hematopoietic potential. Time-lapse imaging and photoconversion experiments demonstrated endothelial-to-hematopoietic transition (EHT) in endocardial cells after 24 h post-fertilization (hpf) [17], coinciding with the incorporation of SHF progenitors into the heart [41]. This process is regulated by *gata5/6* and the *hedgehog* signaling pathway, independent of *etv2* and *scl*, which were previously thought to be master regulators of hematopoiesis, suggesting a new regulatory pathway specific to the endocardium [17]. Furthermore, this study performed scRNA-seq to reveale that endocardium-derived blood progenitor cells are mostly neutrophils, updating the previous recognition that hemangioblasts in the rostral region of the ALPM are a source of myeloid cells, including neutrophils [17]. These results suggest that endocardial hematopoiesis in zebrafish can be considered as a type of primitive hematopoiesis, since it is derived from ALPM and only contributes to myeloid cells.

In contrast, another study reported endocardial hematopoiesis in zebrafish embryos, which is reminiscent of definitive hematopoiesis. Bornhost et al. showed that endocardial cells derived from dorsal aorta endothelial cells undergoing EHT increase after 74 hpf, when cardiac looping is completed [18]. The study also used time-lapse imaging and Kaede photoconversion techniques, along with single-cell RNA-sequencing (scRNA-seq). Although the transcriptional regulatory mechanism of EHT was not determined, the scRNA-seq analysis revealed that most hemogenic endocardial cells express genes involved in promoting hematopoietic stem and progenitor cell (HSPC) fate in the caudal hematopoietic tissue (CHT), known as the definitive hematopoietic site in the zebrafish [18]. These endocardial cell-derived HSPCs are primarily localized in the outer curvature near the outflow tract, an SHF-derived region, and their adhesion is mediated by *itga4* and *vcam1* [18]. Moreover, HSPC production from endocardial cells was suggested to be involved in the maintenance of systemic erythrocyte counts in a compensatory manner [18]. Taken together, the study showed that the endocardium contributes to hematopoiesis both as a tissue source and as a niche, possibly reproducing definitive hematopoiesis.

Although the molecular mechanisms and functional significance of these processes remain to be fully elucidated, the zebrafish endocardium has been shown to harbor hematopoietic programs resembling both primitive and definitive hematopoiesis. Notably, each program shares certain similarities with mammalian endocardial hematopoiesis. Given that the mechanisms underlying endocardial hematopoiesis in mammals remain a topic of ongoing debate, these findings in zebrafish offer a valuable framework for exploring the conserved and divergent aspects of endocardial hematopoiesis across species. As such, insights from zebrafish studies may serve as a foundation for developing new models to better understand the role of the endocardium in mammalian hematopoiesis, shedding light on its evolutionary and developmental significance.

### Endocardial Hematopoiesis in Mouse

In mammals, hemangioblasts are present as yolk sac blood islands, which are functionally analogous to those described in *Drosophila* and zebrafish. These blood islands give rise to both endothelial and hematopoietic progenitor cells, playing essential roles in primitive hematopoiesis and vasculogenesis. Within the embryo proper, however, distinct hemangioblasts with hemo-endothelial biopotency have not been identified. Instead, endothelial cells in regions such as the AGM undergo EHT, acquiring hematopoietic potential and generating hematopoietic progenitor cells.

Interestingly, while cardiogenic progenitors in *Drosophila* and zebrafish emerge from primitive hematopoietic regions such as the cardiogenic mesoderm and ALPM, mammalian cardiogenic progenitors in the lateral plate mesoderm of the embryo proper exhibit a unique feature. During the very early stages of cardiac progenitor specification, these mammalian cardiac progenitors transiently express hematopoietic transcription factors [42], reflecting a potential evolutionary connection between hematopoiesis and cardiogenesis. Although latent hematopoietic potential in cardiac progenitor cells was suggested transcriptionally more than 2 decades ago, bona fide hematopoiesis from the cardiac region has not been examined for a long time. Because of its scarcity and transient nature, hematopoiesis from the heart has long been overlooked, but our close observation and the use of scRNA-seq analysis (GSE126128 [43], GSE76118 [44]), we have captured that expression of hematopoietic transcription factors is maintained in a subset of endocardial cells during cardiac formation [6, 7] The details of the discovery and proof of endocardial hematopoiesis in mice are described below.

The plasticity of endocardial cells has predominantly been examined in relation to their roles in mesenchymal cell differentiation in the cardiac cushion and the formation of coronary endothelial cells. However, their potential to contribute to hematopoiesis remains an underexplored area [45–49]. Endocardial cells, which line the heart’s inner surface and typically exhibit a squamous morphology, undergo a marked transformation during hematopoietic induction. These cells adopt a rounded shape and begin expressing early hematopoietic markers such as *CD41* and *Tal1* [9, 11]. Both our findings and those of others have revealed the presence of endocardial cells expressing hematopoietic markers within specific regions of the mouse embryonic heart, including the outflow tract, atrioventricular canal, and inflow tract [9, 12]. This endocardial hematopoiesis is spatiotemporally coincident with endocardial cushion formation from SHF-derived endocardium, which is regulated by *Nkx2-5* (Fig. 1c) [6, 49, 50]. *Nkx2-5* knockout mice, which fail to develop endocardial cushions and exhibit hypoplastic cardiomyocytes, die during mid-gestation [50, 51]. Importantly, these mice also exhibit severe hematopoietic defects in both the yolk sac and the endocardium, highlighting the multiple roles of *Nkx2-5* in regulating EHT, endothelial-to-mesenchymal transition (EndoMT), and cardiogenesis in mammals [9, 50, 51]. Of note, endocardial hematopoiesis occurs independently of regulation by *Runx1*, suggesting a primitive nature of the endocardial hematopoiesis [9, 13].

The scRNA-seq analysis (GSE76118 [44]) identified two critical pathways involved in *Nkx2-5*-dependent endocardial hematopoiesis: *Notch* signaling and retinoic acid (RA) signaling [10]. Activation of *Notch* signaling in *Nkx2-5*-lineage cells enhanced endocardial hematopoiesis [10]. This finding aligns with *tin/notch* hematopoiesis in *Drosophila* cardiogenic mesoderm [6], suggesting an evolutionarily conserved role for endocardial hematopoiesis. Importantly, activation of *Notch* signaling in *Nkx2-5* lineage cells in *Nkx2-5* null-background mice restored the *Nkx2-5* knockout endocardial phenotype, such as lack of endocardial cushion and endocardial hematopoiesis [10]. This suggests that *Notch* signaling plays a pivotal role in regulating the bilateral differentiation of endocardial cells into mesenchymal and hematopoietic cells by *Nkx2-5/Notch* signaling, highlighting the mutual regulation of cardiac and hematopoietic development. In zebrafish, *notch* signaling also governs endocardial fate decisions, driving endocardial cushion formation by directly upregulating EndoMT genes [52, 53]. Additionally, it regulates EHT in the ventral dorsal aorta, promoting HSC emergence [54]. These findings underscore the conserved role of Notch signaling in shaping endocardial cell fate across species.

The involvement of RA in endocardial hematopoiesis was identified through a ligand-receptor-target gene network analysis, called NicheNet [55] using scRNA-seq data [10]. The enzyme gene *Dhrs3* (*Dehydrogenase/reductase 3*), which regulates the reduction of all-trans retinoic acid (atRA), is one of the most targeted genes in the hemogenic endocardial cell cluster and is significantly downregulated in *Nkx2-5* knockout endocardial cells [10]. Ex vivo hematopoietic colony formation assays revealed that excessive RA signaling impaired the differentiation of hematopoietic progenitors, including macrophages, underscoring the importance of RA suppression via *Dhrs3* expression in endocardial hematopoiesis [10].

Taken together, during mouse cardiogenesis, SHF-derived *Nkx2-5*-lineage endocardial cells undergo EHT via *Notch* signal activation, suggesting conserved mechanisms shared with *Drosophila* and zebrafish. The single-cell analysis has demonstrated the presence and the molecular mechanisms of endocardial hematopoiesis, a rare and transient population that has been overlooked.

### Controversies in Endocardial Hematopoiesis in Mice

Recent studies have raised questions about the hemogenic potential of the mammalian endocardium, offering counterarguments to earlier research suggesting that endocardial cells contribute to hematopoiesis. The study from Liu K et al. conducted comprehensive genetic lineage tracing experiments using various Cre-driver lines, including *Nfatc1-ires-Cre*,* Npr3-CreER*, and *Cdh5-2 A-Cre*, crossed with *Rosa26-LSL-tdTomato* reporters, to trace the fate of endocardial cells during cardiogenesis [15]. These experiments did not detect *Runx1*^+^ endocardial cells, providing no evidence for hemogenic activity in endocardium [15]. Additionally, the group employed an advanced genetic labeling system, the gTCCC (genetic tracing of cell-cell communication) system, which permanently traces heart-specific endothelial cells through intercellular interactions [16]. This approach utilizes genetic markers to trace endothelial populations within the heart, offering a refined method for lineage tracing based on cell-cell communication. The system provides insights into the dynamic behavior of endothelial cells during heart development and function [16]. They utilized the gTCCC system with markers like *Tnnt2* or *Wt1* for sender cells and *Nfatc1* or *Cdh5* for receiver cells, enabling lineage tracing of endocardial and endothelial cells in contact with myocardium or epicardium [16]. Despite its sophistication, the approach failed to identify Runx1^+^ expression in the endocardial cells [16]. Accumulation of data and technological innovation will offer an opportunity to gain insights into the mutual regulation of cardiovascular and hematopoietic differentiation. Further discussions of potential factors underlying these negative results have been explored elsewhere [56].

### Role of Hematopoietic Cells in Cardiac Development

Macrophages are the most abundant hematopoietic cell type in the developing heart. This section summarizes current knowledge about the diverse roles of cardiac macrophages during development. A more detailed review of this topic can be found in our recent article in *Experimental Hematology* [57].

#### Cardiac Cushion Remodeling and Valve Maturation

Our research has shown that hematopoietic cells originating from endocardial cells differentiate into tissue macrophages that localize within the cardiac cushion mesenchyme [9, 10, 13]. These endocardial-derived macrophages exhibit a distinct molecular profile enriched for genes associated with antigen presentation, lysosomal function, and phagosome activity, setting them apart from other tissue-resident macrophage populations [13]. These transcriptomic features were further validated through functional assays, which confirmed their robust phagocytic activity [13]. To investigate the physiological role of endocardial-derived macrophages, we employed genetic models to selectively ablate these cells. By crossing *Nfatc1-Cre* or *Nkx2-5-Cre* mice with *Csf1r*^*flox/flox*^ mice, we achieved targeted deletion of the *Csf1r* (*Colony-stimulating factor 1 receptor*), an essential regulator of macrophage differentiation, specifically in endocardial cells. Mice lacking endocardial-derived macrophages displayed cardiac valve abnormalities, including excessive extracellular matrix (ECM) deposition and increased cellular density, indicating the importance of these macrophages in valve remodeling [10, 13]. Interestingly, despite the recruitment of monocyte-derived macrophages to compensate for the loss of endocardial-derived macrophages, the cardiac valve defects were not resolved [10, 13]. This observation underscores the specialized and non-redundant role of endocardial-derived macrophages in cardiac cushion remodeling and valve formation.

As indicated above, zebrafish studies have demonstrated that hemogenic endocardial cells contribute to neutrophils or HSPCs during development, with minimal contribution to tissue macrophages [17, 18] On the contrary, studies in mice have shown that tissue macrophages in the endocardial cushion and cardiac valve region are one of the major populations of hemogenic endocardial cell derivatives [9, 10, 13]. This difference likely stems from variations in species-specific physiological requirements and developmental processes. Zebrafish valve formation is less dependent on extensive remodeling, as their valve mesenchyme produces thin, simple valve structures [58, 59]. In contrast, mammalian valve development requires significant ECM remodeling to create the complex and durable architecture necessary for managing higher circulatory pressures [60, 61]. This underscores the crucial role of endocardial-derived macrophages in mammalian cardiac development, particularly for reshaping cardiac cushions into structurally robust valves suited to the mechanical demands of the mammalian circulatory system.

Overall, these conflicting data emphasize the need for further investigation of the physiological significance of endocardial hematopoiesis. It is likely that hematopoietic endocardial cells differentiate primarily into macrophages and contribute to endocardial cushion remodeling, an adaptation in the morphogenesis of the heart that is unique to mammals.

#### Coronary and Lymphatic Vessel Remodeling

Although not limited to endocardial origin, hematopoietic cells play a crucial role in coronary vessel formation, as evidenced by analysis of mice deficient in hematopoietic progenitor cells, including *Runx1* knockout mice [62]. Ventricular explants from *Runx1*-null embryos exhibit a marked reduction in CD31^+^ endothelial sprouting and network formation, independent of hemodynamic factors [62]. Notably, co-culture of *Runx1*-null explants with CD45^+^ hematopoietic cells rescued endothelial sprouting, indicating that hematopoietic cells provide essential signals for coronary vascularization [62]. Building on this, Ccr2-negative fetal tissue macrophages have been identified as key regulators of coronary vessel remodeling [63]. Their absence results in excessive microvascular branching and a reduction in smooth muscle-coated coronary arterioles [63]. These vascular defects are attributed to impaired epithelial-mesenchymal transition (EMT) of epicardial-derived cells, a process essential for the differentiation of vascular smooth muscle cells, perivascular fibroblasts, and stromal fibroblasts—critical components in stabilizing the coronary vasculature [64, 65]. *Runx1* knockout embryos also exhibit defects in epicardial EMT [62], further supporting the idea that hematopoietic progenitors and fetal tissue macrophages play pivotal roles in coronary vessel remodeling during cardiac development.

Fetal tissue macrophages are found to be also important for the development of lymphatic vessels in the heart [66]. In this study, scRNA-seq analysis identified that *Cx3cr1*^+^*Csf1r*^+^ tissue macrophages appear in the mouse embryonic heart as early as embryonic day 10.5 (E10.5), and that these macrophages co-localize with coronary and lymphatic vessels once they begin to form [66]. Moreover, macrophage depletion with Pu.1 knockout and Cx3cr1-CreER; R26-DTA mice was shown to impair lymphatic vessel growth and branching, highlighting the critical contribution of fetal tissue macrophages to cardiac lymphangiogenesis [66]. This is likely a function of Vegfc released by macrophages [67].

In summary, hematopoietic progenitor cells and fetal macrophages play a crucial role in the formation of coronary arteries and lymphatic vessels in the developing heart. Thus, it is indicating that hematopoietic cells do not just pass through the vasculature, but are actively involved in the morphogenesis of the vasculature.

#### Cardiomyocyte Maturation

A recent study revealed that macrophages derived from human embryonic stem cells (hESCs) can integrate into human cardiac microtissues, adopting transcriptional profiles resembling those of yolk sac and fetal cardiac tissue macrophages [68]. These cells express markers such as TIMD4, LYVE1, and FOLR2, while lacking CCR2 expression [68], which are established markers of yolk sac-derived tissue macrophages [69, 70]. One key mechanism through which macrophages support cardiomyocyte maturation is efferocytosis, the clearance of apoptotic cells and cellular debris [68]. Additionally, macrophages contribute to metabolic remodeling in cardiac tissue by modulating mitochondrial dynamics [68]. By facilitating the removal of dysfunctional mitochondria from cardiomyocytes, they help maintain cellular energy homeostasis and enhance contractile function [68]. Although the precise signaling pathways governing macrophage-driven cardiac maturation remain incompletely understood in vivo, this study provides valuable insights into how macrophages influence early cardiac maturation by regulating cardiomyocyte growth, sarcomeric organization, and metabolic adaptation [68]. This study underscores the idea that macrophages are not merely immune scavengers but active regulators of cardiac development and function.

## Conclusions

Mammalian endocardial cells have been identified as a novel fetal hematopoietic site, challenging the longstanding belief that embryonic hematopoiesis, comprising multiple waves, occurs exclusively in the yolk sac and AGM region. As highlighted in this review, the hemogenic potential of endocardial cells aligns with evolutionary conservation. Notably, single-cell analysis helped to elucidate this phenomenon. This is because single-cell analysis allows researchers to unravel cellular heterogeneity, identify rare cell populations, reconstruct differentiation trajectories, and explore complex regulatory networks. The identification of hemogenic endocardial cells exemplifies the necessity of such high-resolution approaches in developmental biology.

This paradigm shift invites a reevaluation of the fundamental principles of embryonic hematopoiesis and cardiogenesis. Future studies are anticipated to elucidate how endocardial-derived hematopoietic cells contribute to heart development and pathology, as well as their potential applications in regenerative medicine. Comparative research with other model organisms will further enhance our understanding of evolutionary conservation, ensuring that this concept continues to shape both the hematology and cardiac development fields.

## Key References

- Liu N, Kawahira N, Nakashima Y, et al. (2023) Notch and retinoic acid signals regulate macrophage formation from endocardium downstream of Nkx2-5. Nat Commun 14:5398.
-
  - The findings in this study utilize scRNA-seq analysis to elucidate the molecular mechanisms of endocardial hematopoiesis in mice.
- Gurung S, Restrepo NK, Sumanas S (2024) Endocardium gives rise to blood cells in zebrafish embryos. Cell Rep 43:113736.
-
  - The findings in this study identified endocardial hematopoiesis in zebrafish around 24 hpf, primarily giving rise to neutrophils.
- Bornhorst D, Hejjaji A V., Steuter L, et al. (2024) The heart is a resident tissue for hematopoietic stem and progenitor cells in zebrafish. Nat Commun 15:7589.
-
  - The findings in this study identified endocardial hematopoiesis in zebrafish concurrently with definitive hematopoiesis, contributing to the formation of HSPCs.

## Data Availability

No datasets were generated or analysed during the current study.
